# Care of women and application of the principle of informed consent to interventions during birth in the COVID-19 pandemic period

**DOI:** 10.18332/ejm/186069

**Published:** 2024-05-01

**Authors:** Alina Liepinaitienė, Izabelė Bujaitė, Aurimas Galkontas, Vaidas Jotautis, Audrius Dėdelė

**Affiliations:** 1 Department of Environmental Sciences, Faculty of Natural Sciences, Vytautas Magnus University, Kaunas, Lithuania; 2 Faculty of Medicine, Kauno kolegija Higher Education Institution, Kaunas, Lithuania; 3 Faculty of Public Health, Academy of Medicine, Lithuania University of Health Sciences, Kaunas, Lithuania

**Keywords:** informed consent, COVID-19 pandemic, maternity care

## Abstract

**INTRODUCTION:**

In the early phases of the COVID-19 pandemic, inadequate intrapartum care protocols were in place. Many organizations have responded promptly and recognized the importance of adherence to appropriate guidelines. The International Confederation of Midwives issued an official statement on 29 March 2020, which states that every woman has the right to information, to give consent, to refuse consent, and to have her choices and decisions respected and upheld. No research has been conducted in Lithuania to reveal the care of women who gave birth during the COVID-19 pandemic and the application of informed consent to interventions.

**METHODS:**

This study is quantitative of cross-sectional design. An anonymous questionnaire survey method was used. One hundred fifty-two women who gave birth in Lithuania during the COVID-19 pandemic (March 2020 – May 2022) and had COVID-19 infection during childbirth, participated in the study. Statistical data analysis was performed.

**RESULTS:**

During the COVID-19 pandemic, women's care was characterized by always or almost always adequate information from health professionals on all issues to minimize the stress of new procedures necessitated by the COVID-19 pandemic and allowing them to stay with newborns as long as possible. The application of the principle of informed consent to interventions during the COVID-19 pandemic was not always applied to the performance of transvaginal examination manual compression of the uterine fundus to facilitate the expulsion period.

**CONCLUSIONS:**

Most women said that they were properly informed by healthcare professionals about all questions related to the new procedures that became necessary due to the COVID-19 pandemic and felt included in their own choice. However, mothers felt the need of relatives during childbirth, and consent was often not asked for vaginal examination.

## INTRODUCTION

On 11 March 2020, the World Health Organization (WHO) announced the spread of the coronavirus (COVID-19) outbreak to other countries^1^. As the virus continued to spread around the world, every healthcare institution faced new challenges in organizing the provision of services to patients during quarantine. Especially in the early phases of the COVID-19 pandemic, inadequate birth care protocols were used in many situations, leading to increased medicalization. In a review of 108 cases published in English and Chinese up to 4 April, the cesarean section rate in pregnant women who contracted COVID-19 was 92%^2^. Human rights violations were also recorded during the pandemic^3^. Lazzerini et al.^4^ surveyed 21027 women who had given birth from various European countries, and the results showed that 4315 (23.9%) women experienced disrespect, and 2256 (12.5%) respondents said they were insulted. Many organizations have been quick to respond and recognize the importance of complying with relevant requirements in relation to the provision of maternity care^4^. On 30 March 2020, the Lithuanian Society of Obstetricians and Gynecologists published recommendations related to COVID-19 infection and pregnancy. The International Confederation of Midwives emphasized, in an official statement issued on 29 March 2020, that every woman has the right to receive information from healthcare specialists, to give consent to procedures or to refuse, so that their decisions are respected and supported.

In healthcare institutions, an integral part of everyday life is not only to provide help, treatment, and care for patients but also to familiarize the patient with possible risks, performed procedures, and alternative methods of treatment. Informed consent is defined as verbal or written consent of the mother/family member/relative (after being informed and understanding the purpose, benefits, and potential risks of the tests and procedures explained by healthcare providers) before any examination or procedure^5^. Although the requirement of informed patient consent in the provision of healthcare has been established for more than a decade, medical personnel do not always ask for consent for the procedure. In the Czech Republic, 50% of midwives, 46% of doulas, and other healthcare professionals (2%) were interviewed. The aim was to find out whether the midwife or the doctor explained to the woman why it is important to perform a vaginal examination. The obtained results showed that 46% of respondents said that they did not explain the importance of the examination, while only 21% of respondents answered yes, always. Also, 51% said that doctors in the hospital would never ask a woman’s permission for an examination, and 15% of research participants indicated that they would inform the woman about the actions they plan to perform^6^.

During the global pandemic, there was much uncertainty in the work of healthcare institutions, so women may not have been fully informed about the procedures. Consent was not requested before they were performed, but no research has been conducted in Lithuania to reveal the care of women who gave birth during the COVID-19 pandemic and the application of the principle of informed consent to interventions. To understand the problem of the theme, the aim of the study was presented, which is to determine the care of women who gave birth during the COVID-19 pandemic and the application of the principle of informed consent to interventions.

## METHODS

### Study design and collection of data

In order to reveal the care of women who gave birth during the period of the COVID-19 pandemic and the application of the principle of informed consent to interventions, a quantitative study with a cross-sectional design was carried out. An anonymous questionnaire survey method was used. Together with the coordinators of the midwifery care project in Lithuania from various countries, a questionnaire was developed, which aimed to investigate the readiness (participating in maternity preparing lectures, father involvement in the preparation of childbirth, etc.), quality (medical staff informed consent, information in time and environment, etc.) and resilience (mental health) of maternal and newborn healthcare services during the COVID-19 pandemic.

The research data collecting period was 1 November – 10 December 2022; 152 women who gave birth in Lithuania during the COVID-19 pandemic (and had COVID-19 infection during childbirth) participated in the study.

### Ethics

The study followed basic ethics principles. Subjects could refuse to participate in the study. Adults who voluntarily agreed to fill in a questionnaire participated in the study. The respondents were given information about the purpose, nature of the research, and the possibility of getting acquainted with the results of the research in the preamble of the questionnaire. They were informed that the anonymity and confidentiality of the participants would be preserved without collecting any personal data (i.e. name, surname, personal identification number, etc.), and the study data would be presented in a summarized way.

### Participants

The research participants were selected by convenience sampling. All women who met the following criteria were invited to participate in the study: pregnant women who agreed to participate in the study; women aged ≥18 years who had given birth; women who had given birth and understood and spoke Lithuanian; women who gave birth during the COVID-19 pandemic; had COVID-19 during childbirth; participated in the ongoing project and answered the questions of the created questionnaire. Women who did not meet the following criteria were excluded from the study: pregnant women who did not agree to participate in the study; women who had given birth and were aged <18 years; women who did not understand or spoke Lithuanian; women who gave birth outside the period of the COVID-19 pandemic (before 1 March 2020 and after 1 May 2022).

Descriptive statistics were used through calculations of frequencies and percentages.

## RESULTS

A total of 152 women who gave birth during the COVID-19 pandemic (and had COVID-19 during childbirth) participated in the study. Most of the respondents were aged 25–30 years (32.2%, n=49). The data obtained show that for the majority of respondents (74.3%, n=113), labor started spontaneously, and for 11.8% (n=18) labor was induced. An emergency cesarean section during labor was performed in 11 women (7.2%), and an emergency cesarean section was performed before the onset of labor in 1 (0.7%) participant. Instrumental delivery using vacuum extraction or forceps was performed in 2 mothers (1.3%) and 4.6% (n=7) women underwent elective cesarean section.

Most of the respondents (91.4%, n=139) noted that an obstetrician was present during the birth, and more than half (63.8%, n=97) of the women indicated that an obstetrician-gynecologist was present during their birth; 7.9% (n=12) of respondents noted that the healthcare professionals who attended the birth did not introduce themselves, so they did not know which professionals attended the birth.

In order to learn about maternity care during the COVID-19 pandemic, questions were asked about the requirements that were introduced during the pandemic. Respondents were asked about the possibility of staying with their loved one until the woman felt the need. The results obtained show that 46.1% (n=70) of respondents indicated that the relative could never or almost never stay until the woman felt the need. However, 34.2% (n=52) of mothers felt that a relative could always or almost always stay with the woman.

Just under half (49.3%, n=75) of women who completed the questionnaire said that healthcare professionals provided adequate information on all issues to minimize stress related to new procedures necessitated by the COVID-19 pandemic. However, 19.7% (n=30) of respondents indicated that they were not properly informed about the new procedures.

According to the results of the study, 67.8% (n=103) women said that healthcare professionals always or almost always provided help immediately when the woman needed it, while the remaining (32.2%, n=49) women felt that help was sometimes provided. Respondents were also asked whether they received attention during the appropriate time when they arrived at the hospital (waiting time, appointments for further care, hospitalization, discharge to home, etc.) and 66.4% (n=101) answered that they received attention at the right time, but 6.6% (n=10) felt that it was not done in a timely manner.

Of the 152 interviewed women, 73 (48.0%) answered that after the onset of strong and regular contractions, the specialists who participated in the birth allowed them to move freely (get out of bed, walk, exercise), 37 (24.3%) of the mothers who participated in the study indicated that they were only allowed to get out of bed or change position sometimes, and 42 (27.6%) noted that they were asked to stay in bed all the time, lie on their back or turn on their left or right side.

The women who participated in the study were asked whether the staff wore and used personal protective equipment (masks, gloves, etc.) properly during each visit. More than half (63.8%, n=97) indicated that personal protective equipment was always or almost always worn, 29.6% (n=45) believed that the equipment was sometimes worn and used properly, and 6.6% (n=10) indicated that personal protective equipment was never or almost never used.

To find out the application of the principle of informed consent during childbirth, women were asked whether healthcare professionals asked whether the mother in labor consented to the vaginal examination. In all, 44.1% (n=67) answered that consent was not requested, but 29.6% (n=45) respondents indicated that consent was obtained before performing the vaginal examination.

More than half (61.2%, n=93) of mothers answered that health facility workers did not inform them about possible dangerous clinical signs such as profuse vaginal bleeding, difficulty urinating, and difficulty breathing, but 38.8%. (n=59) said that they were fully informed. They were also asked about possible clinical signs dangerous to the newborn (profuse sweating, tremors, difficulty breathing, shivering, or neonatal jaundice). The data obtained show that the majority (67.1%, n=102) were also not fully informed about possible symptoms, but 32.9% (n=50) of women were given detailed explanations of the symptoms that may occur.

Of respondents, 50.0% (n=76) stated that the health facility staff sometimes included them in the choice of the provided care/treatment (explained precisely, respected autonomy, asked for preferences or opinions), and 7.9% (n=12) said that they were never or almost never involved in making a choice. It is good that almost half (42.1%; n=64) of the study participants indicated that they were always or almost always involved in independent choice.

Most (60.5%, n=92) of the women who participated in the study assessed their experience in the hospital positively after taking into account all previous considerations, and 28.3% (n=43) of mothers reported a very positive experience. Only 10.5% (n=16) rated it as a negative experience, and only 0.7%. (n=1) felt that their experience was very negative.

## DISCUSSION

During the global pandemic, women’s childbirth experience may have been affected by various difficulties and uncertainties. Due to the restrictions of COVID-19, special action plans have been prepared for inpatient facilities providing obstetric services, following the orders and legal acts of the Ministry of Health. In order to determine maternity care during the COVID-19 pandemic and the application of the principle of informed consent to interventions, a study was conducted with women from various cities in Lithuania.

The results of the study showed that 46.1% of respondents in Lithuania indicated that the relative could never or almost never stay until the woman felt the need. However, 34.2% of the women who participated in the study felt that the relative could always or almost always stay with the woman. Meanwhile, in a study conducted by Sanders and Blaylock^7^, women who gave birth during the COVID-19 pandemic indicated that they felt anxious because of hospital-imposed restrictions on the presence of a partner during the birth: *‘These restrictions caused a lot of anxiety for me and my partner because it was our first baby and we wanted to do everything together. In the hospital, there were restrictions that partners were not admitted until the cervix was 7 cm dilated’*. This can be attributed to the fact that the presence of relatives can give peace of mind to patients during childbirth, and the COVID-19 pandemic not only caused anxiety due to the unknown disease and various restrictions but also due to the inability of relatives to participate in childbirth.

On 3 March 2020, Favre et al.^8^ provided recommendations for pregnant women with suspected SARS-CoV-2 infection, which suggested that newborns of infected women should be isolated for at least 14 days or until viral shedding subsides, during which time direct breastfeeding is not recommended. However, in our research, the majority of women who participated (87.5%) said that they were allowed to stay with the baby as long as they wanted while in the hospital after giving birth. However, 12.5% of respondents disagreed with this statement. Of course, the separation of the newborn from the mother could also be influenced by the poor condition of the newborn, cesarean section operation, or other emergency situations.

Asefa et al.^9^, in a study conducted by one of the midwives interviewed, stated that due to the lack of staff, challenges of social restriction, and increased requirements for disinfection, women in labor had to stay in the beds or wards where they were admitted, not move and could not use shared toilets or showers. In our study, 27.6% of women also noted that after the onset of strong and regular contractions, the professionals attending the birth asked them to stay in bed the whole time, lie on their backs, or turn on their left or right side. It can be assumed that some women were not allowed to move freely during childbirth, but a larger study is needed to find out the reason why women were not able to move.

In order to reveal the application of the principle of informed consent to interventions, a study in Lithuanian, it was enquired whether healthcare professionals asked whether the mother consented to the vaginal examination; 44.1% answered that consent was not requested. Similar results were obtained by Adu-Bonsaffoh et al.^10^ in a study of 1430 women; 842 women (58.9%) reported that vaginal examination was performed without consent. It seems that the obtained data show that specialists do not always ask for consent to perform this procedure, so a larger study would be needed to find out the reasons.

In a Czech study, Begley et al.^6^ found that 45% of surveyed medical workers said that the Kristeller method is used in about a quarter of all births, and 31% indicated in approximately half of all births. Sixty-three percent of respondents said that specialists would explain to the woman why, in their opinion, it is necessary to do this, and only 18% answered that they would ask the woman’s permission^6^. In our research, 31.6% (n=48) of respondents indicated that, in order to facilitate the birth of the newborn, healthcare professionals also did not ask for consent to press the abdomen with their hands to help with the childbirth. The obtained data are not encouraging, as it can be assumed that this method can still be used frequently during childbirth.

Despite the changes in healthcare institutions due to the COVID-19 pandemic, the majority of the women who participated in the study evaluated their experience in the hospital positively.

### Strengths and limitations

The study has some limitations. The data collected were based on self-reports, which might be subject to reporting bias. Additionally, the study’s generalizability to all women during the COVID-19 pandemic may be limited. Furthermore, since the largest hospital in Lithuania did not participate in the study, the results may not be representative of the entire population of women in the country. Despite these limitations, this study is significant as it sheds light on how the COVID-19 pandemic has impacted women in Lithuania, which is relatively new information.

## CONCLUSIONS

The majority of women who gave birth during the COVID-19 pandemic said that they always or almost always received adequate information from healthcare professionals on all issues to minimize stress related to new procedures necessitated by the COVID-19 pandemic, received timely attention, and allowed to stay with the newborns as long as they wanted. However, it was noted that it was not always possible for a loved one to stay during childbirth as long as the mother felt the need. Some women also indicated that they were asked to stay in bed for part or all of the time, to lie on their backs, or to turn on their left or right sides.

The majority of women who gave birth during the COVID-19 pandemic indicated that they always or almost always felt included in their own choice and that the staff of medical facilities protected their privacy, and when they asked for pain relief during childbirth, it was provided. However, more than one in four women said that they were not asked for consent to perform a vaginal examination to facilitate the birth of a newborn; healthcare professionals did not ask for the woman’s consent to press the abdomen with their hands to help give birth; and did not inform about possible dangerous clinical signs for the mother and the newborn.

The implication for future pandemics or health issues for all people is to give clear, accurate, and timely information related to the patient’s health, not to leave the woman alone, and to ensure the updating of obstetric recommendations.

## Data Availability

The data supporting this research are available from the authors on reasonable request.
